# Home-based intervention to test and start (HITS) protocol: a cluster-randomized controlled trial to reduce HIV-related mortality in men and HIV incidence in women through increased coverage of HIV treatment

**DOI:** 10.1186/s12889-019-7277-0

**Published:** 2019-07-19

**Authors:** T. Mathenjwa, H.-Y. Kim, T. Zuma, M. Shahmanesh, J. Seeley, P. Matthews, S. Wyke, N. McGrath, B. Sartorius, H. M. Yapa, O. Adeagbo, A. Blandford, A. Dobra, T. Bäernighausen, F. Tanser

**Affiliations:** 1 Africa Health Research Institute, Durban, South Africa; 2 Institute for Global Health, University College London, London, United Kingdom; 3 London School of Hygiene and Tropical Medicine, London, United Kingdom; 4 University of Glasgow, Glasgow, United Kingdom; 5 University of Southampton, Southampton, United Kingdom; 6 Faculty of Infectious and Tropical Diseases, London School of Hygiene and Tropical Medicine, London, United Kingdom; 7 The Kirby Institute, University of New South Wales, Sydney, Australia; 8 UCL Interaction Centre, University College London, London, United Kingdom; 9 University of Washington, Washington, USA; 10 University of Heidelberg, Heidelberg, Germany; 11 Department of Sociology, University of Johannesburg, Johannesburg, South Africa; 12 School of Nursing and Public Health, University of KwaZulu-Natal, Durban, South Africa; 13 Centre for the AIDS Programme of Research in South Africa (CAPRISA), University of KwaZulu-Natal, Durban, South Africa; 14 Research Department of Infection & Population Health, University College London, London, United Kingdom

**Keywords:** HIV, Home-based HIV testing, Financial incentives, Counselling, Linkage to care

## Abstract

**Background:**

To realize the full benefits of treatment as prevention in many hyperendemic African contexts, there is an urgent need to increase uptake of HIV testing and HIV treatment among men to reduce the rate of HIV transmission to (particularly young) women. This trial aims to evaluate the effect of two interventions - micro-incentives and a tablet-based male-targeted HIV decision support application - on increasing home-based HIV testing and linkage to HIV care among men with the ultimate aim of reducing HIV-related mortality in men and HIV incidence in young women.

**Methods/design:**

This is a cluster randomized trial of 45 communities (clusters) in a rural area in the uMkhanyakude district of KwaZulu Natal, South Africa (2018–2021). The study is built upon the Africa Health Research Institute (AHRI)‘s HIV testing platform, which offers annual home-based rapid HIV testing to individuals aged 15 years and above. In a 2 × 2 factorial design, individuals aged ≥15 years living in the 45 clusters are randomly assigned to one of four arms: i) a financial micro-incentive (food voucher) (*n* = 8); ii) male-targeted HIV specific decision support (EPIC-HIV) (*n* = 8); iii) both the micro incentives and male-targeted decision support (*n* = 8); and iv) standard of care (*n* = 21). The EPIC-HIV application is developed and delivered via a tablet to encourage HIV testing and linkage to care among men. A mixed method approach is adopted to supplement the randomized control trial and meet the study aims.

**Discussion:**

The findings of this trial will provide evidence on the feasibility and causal impact of two interventions - micro-incentives and a male-targeted HIV specific decision support - on uptake of home-based HIV testing, linkage to care, as well as population health outcomes including population viral load, HIV related mortality in men, and HIV incidence in young women (15-30 years of age).

**Trial registration:**

This trial was registered on 28 November 2018 on, identifier https://clinicaltrials.gov/.

## Background

Early initiation of antiretroviral therapy (ART) has the dual benefit of increasing life expectancy for people living with HIV and preventing onward HIV transmission to uninfected individuals [1, 2]. In sub-Saharan Africa, the widespread scale-up of ART has reduced HIV-related deaths by almost half and led to substantial declines in new HIV infections [1]. For example, in a rural area in Northern KwaZulu-Natal, South Africa, a 1% increase in community-level ART coverage was associated with a 1% reduction in the individual risk of HIV acquisition [2]. However, the overall HIV incidence rates remain high in this setting, and young women are disproportionately at a risk of HIV infection [3]. HIV-related mortality among men also remains comparatively high despite the mass provision of free ART in public health care facilities [4, 5]. These two challenges to current HIV programs – continued high HIV incidence in women and comparatively high HIV-related mortality in men – are inextricably linked. The key contributing factors are that men are less likely to test for HIV and far less likely to link to care if tested HIV positive [6], putting them at a greater risk of HIV-related death, as well as HIV transmission to their sexual partners.

To realize the full benefits of treatment as prevention, it is urgent to increase uptake of HIV testing and HIV treatment among men who are living with HIV and at high risk of transmitting HIV to their sexual partners. Behavioural economics suggests that relatively small financial incentives can be used to “nudge” individuals towards healthy behaviours [7], particularly simple behavioural changes, such as agreeing to a rapid HIV test or making clinic appointments. Incentives have been shown as effective in increasing one-off behaviours, such as vaccinations [8], tuberculin screening testing [9], circumcision [10], or uptake of preventative health counselling in low- and middle-income countries [11]. However, the causal impact of one-off financial incentives for HIV testing and linkage to care on long-term population-level benefits such as reducing HIV incidence has not been established.

On the other hand, long-term linkage to care involves repeated trips to the clinic which require ongoing intrinsic motivation. Yet, financial incentives tend to focus on short-term behavioural changes and operate via extrinsic motivation, which could potentially crowd out intrinsic motivation [12, 13]. The benefits of testing and early initiation of ART are also not widely understood or accepted by many men, often delaying HIV testing and linkage to care after diagnosis [14]. In the treatment as prevention (TasP) trial (ANRS 12249) conducted in KwaZulu-Natal, South Africa, the majority of men who did not consent for HIV testing reported feeling healthy as a reason for being reluctant to test [15]. These barriers to test and link to care are usually based on lack of information, misinformation or lack of salience of the information. Providing decision support in the form of experiential information that describes the options available, allows clarification of personal values, and the need to make specific decisions (to test or link) explicit [16] could increase intrinsic motivation. Also, given the lower level of testing and linkage to care among men compared to women, decision support for HIV testing and linkage to care needs to be tailored, flexible and gender sensitive to reflect existing values and norms of men and women, separately. Complementing financial incentives with gender specific HIV counselling could be an effective strategy to increase both intrinsic and extrinsic motivation to test and link to HIV care.

We hypothesize that provision of micro-incentives and male-targeted HIV-specific decision-support (implemented via an android tablet-based application) would enable people to make more informed choices and thus increase uptake of home-based HIV testing and linkage to care, leading to reduction in population viral load and HIV-related mortality among men and ultimately lower HIV incidence in young women. To test this hypothesis, we designed a factorial cluster randomised controlled trial for implementation in the uMkhanyakude district of KwaZulu-Natal, South Africa.

## Methods/design

### Study design

**Fig. 1:**
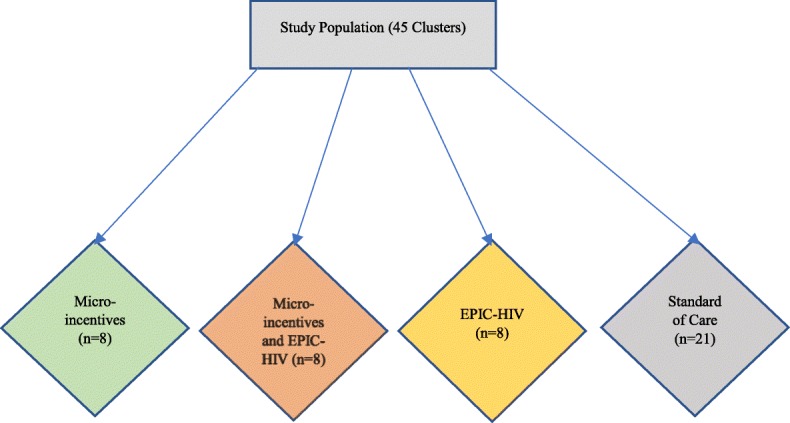
Allocation of the two interventions in four strata across 45 communities

The trial is delivered in a four strata using a 2 × 2 factorial design to compare the effectiveness of two interventions, micro-incentives and male-targeted HIV-specific decision-support, called EPIC-HIV (Empowering People through Informed Choices for HIV), in 45 clusters (week blocks) using the Africa Health Research Institute (AHRI)‘s HIV surveillance platform (Fig. 1). The four strata are Arm 1 micro-incentives; Arm 2 both micro-incentives and EPIC-HIV (male-targeted HIV decision-support); Arm 3 EPIC-HIV (male-targeted HIV decision-support); and Arm 4 standard of care (SoC).The study protocol follows the Standard Protocol Items: Recommendations for Intervention Trials (SPIRIT) [17] and the Consolidated Standards of Reporting Trials (CONSORT) statement for reporting RCTs [18].

The study is registered at the National Institute for Health’s ClinicalTrials.gov. Enrolment started in February 2018 and follow up will be completed in December 2021.

### Participants

#### Inclusion criteria

Individuals are eligible if they are aged 15 years or older and a resident member of the of households within the AHRI HIV Surveillance southern area, agree to participate in the annual HIV surveillance and willing to give written informed consent for trial participation. Both males and females are eligible to receive the micro-incentive component of the intervention but only males are eligible to receive EPIC-HIV (male-targeted HIV decision-support).

#### Exclusion criteria

Individuals are not eligible to receive any of the HITS interventions if they have refused to participate in the AHRI HIV surveillance or report to be already on ART. Individuals mentally or physically challenged to provide consent are also excluded.

### Study setting

**Fig. 2:**
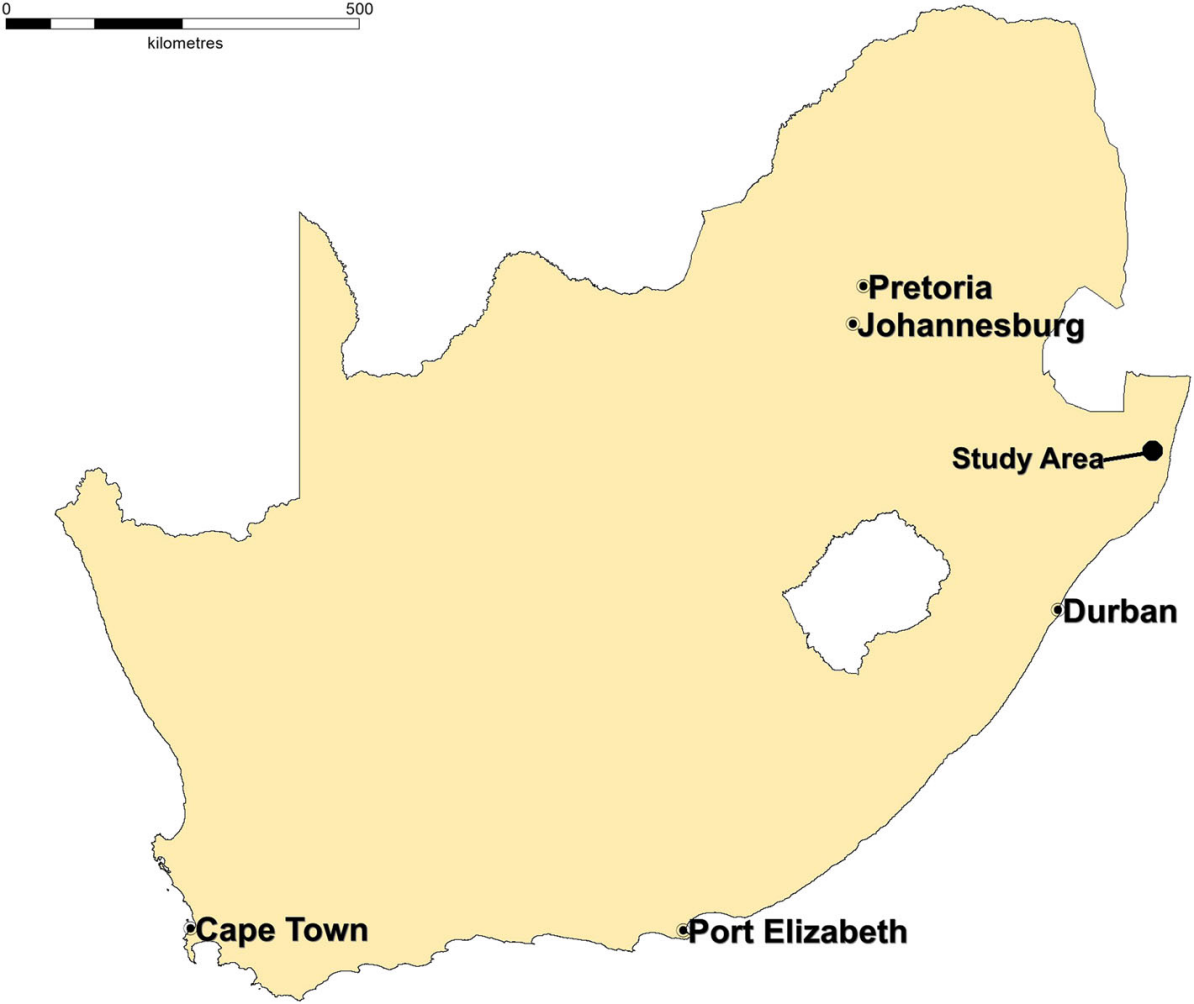
Location of the study area in South Africa: Taken from another source - Tanser et al. 2008 [19]. Not under copyright

The trial is conducted in the Hlabisa sub-district of the uMkhanyakude district located in northern KwaZulu-Natal South Africa between 2018 and 2021 (Fig. 2). The area is predominantly rural and has recorded one of the highest population HIV prevalence estimates globally [20]. In this sub-district, AHRI has one of the largest population-based Demographic and HIV surveillance since 2000. The surveillance covers 432 km^2^ geographic area over approximately 100 000 individuals and 60 000 residents at any given time. Trained field-workers visit all households in the surveillance area and interview a key resident informant annually. The household survey records demographic components of households including size, composition, fertility, migration, mortality, and nuptiality. The HIV surveillance is annually conducted on all resident individuals aged 15 years or older to collect sexual behaviour and general health data and dried blood spots (DBS) samples for anonymized HIV testing [21]. The fieldwork visits are carried out in systematic cycles of 45 "week blocks". A week block is a workload-equivalent area developed using a GPS based methodology [22]. AHRI works closely with the local Department of Health in a comprehensive HIV Treatment and Care Programme, decentralized to the 17 primary health care clinics in the sub-district. HIV testing and treatment are available at all 17 primary health care clinics and the district hospital. Since 2017, AHRI has introduced home-based rapid HIV testing as part of the annual HIV surveillance.

### Interventions

**Fig. 3:**
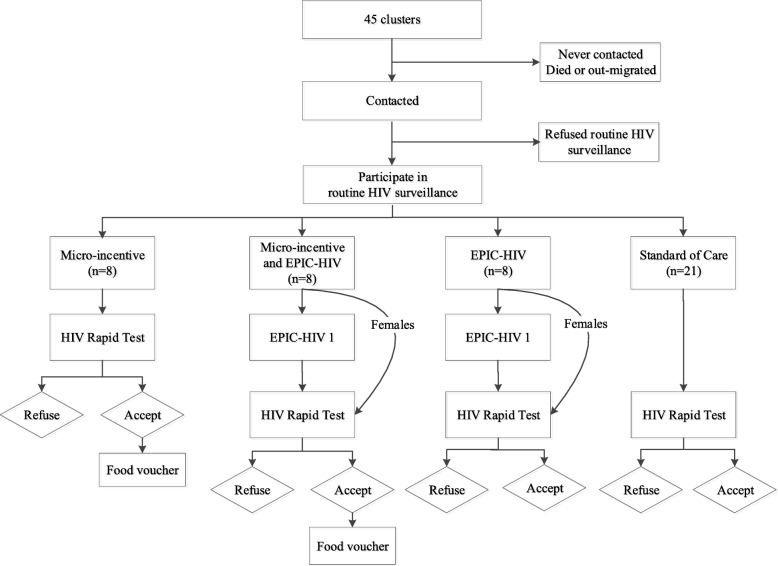
Study flow - 1st stage: interventions at the time of home-based HIV testing offer

**Fig. 4:**
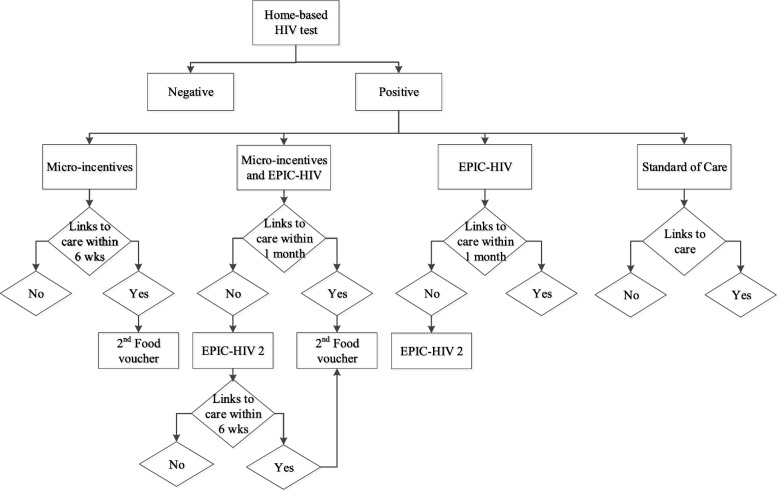
Study flow – 2nd stage: interventions at the time of linkage to care (Arm 1 and 2 for micro-incentives) or 1 month after HIV-positive test if not linked to care (Arm 2 and 3 for EPIC-HIV 2)

The two interventions, micro-incentives and the male-targeted HIV-specific decision support, are offered in a two-stage scheme. The first stage is aimed at encouraging HIV testing (Fig. 3), while the second stage is aimed at encouraging linkage to care if tested HIV positive (Fig. 4). Micro-incentives are in the form of a R50 food voucher (redeemable in a local supermarket) conditional on undergoing a home-based rapid HIV test. If diagnosed with HIV and present for HIV care in one of the local Department of Health clinics servicing the program area within 6 weeks of the HIV test, participants receive a second R50 food voucher.

The male-targeted HIV-specific decision-support is implemented via a tablet-based application called EPIC-HIV. EPIC-HIV development was guided by a combination of human behaviour change theory and person-based intervention design [23] and human computer interaction techniques. The development process (described elsewhere in more detail) included three phases: 1) a thorough review of literature was conducted to synthesize key facilitators and barriers of HIV care and management among men in South Africa; 2) the review informed content, design features and format of the application, and the app was iteratively prototyped; 3) the content was evaluated with the community advisory board, and usability testing and in-depth interviews were conducted with representative users at every iteration to establish understanding of content and usability issues. The content is rooted in local narratives and provided in a form of experiential information to increase risk perception, salience and likelihood of response [24, 25]. EPIC-HIV makes the decision to test or link to care explicit by providing experiential information about what various outcomes might be like (imagined future). It further supports three basic human ‘needs’ as defined by the self-determination theory - the need for autonomy, competence, and relatedness [26]. The app is available in two versions (EPIC HIV-1 and EPIC HIV-2). EPIC-HIV 1 is offered to men at the point of HIV test offer prior to HIV counselling and testing to support them  when choosing whether or not to test for HIV. If participants are diagnosed with HIV but do not present in the local Department of Health clinic within a month of the positive HIV test, a study tracker team re-visit participants at home and offer EPIC-HIV 2, which is designed to address barriers to seek HIV treatment and encourage them to link to HIV care.

Male residents in the clusters that receive both interventions are offered EPIC-HIV 1 prior to HIV testing, a food voucher conditional on undergoing a rapid HIV test, and a second food voucher when they link to care within 6 weeks of receiving an HIV positive test. If they do not link to care within a month of the positive HIV test they become eligible for EPIC-HIV 2. Female residents in these clusters are eligible to receive the food voucher only. Residents in the control clusters receive standard of care where they are offered the home-based HIV rapid test, and if diagnosed with HIV, referred to the clinic for linkage to HIV care.

### Outcomes

The goal of the trial is to establish whether the provision of micro-incentives and a male-targeted HIV-specific decision-support app will increase uptake of HIV testing and linkage to HIV care thus, ultimately reducing HIV-related mortality in men and population-level HIV incidence in young women. The trial’s primary endpoints are to measure the impact of the interventions at both individual and population-levels as follows:

1. HIV testing uptake at point of test offer in men
2. HIV treatment linkage in men at 1-year
3. Population-level HIV viral suppression in men after 1-year
4. Population-level HIV-related mortality in men after 3-years
5. Population-level HIV incidence in young women (15–30 years of age) after 3-years

Specifically, the study compares differences in the percentage that received the rapid HIV test at home among those offered the test in men among the intervention arms compared to the SoC arm. The study will also compare the percentage who visited study clinics and initiated ART in men at 1-year after the home-based HIV-positive test, as well as prevalence of detectable viremia (detection limit of > 1550 copies/ml) obtained from the DBS in men. Changes in the number of HIV-related deaths and mortality rates in men and changes in the number of new HIV infections and HIV incidence rates in young women 3 years after the home-based HIV test will be compared across the arms. Secondary outcomes that will be measured in the study are shown in Table 1.

**Table 1:** Secondary outcomes of the study

No	Secondary outcome	Description	Time frame
1	HIV testing uptake at point of test offer in women	% received rapid HIV test at home among those offered the test in women	Baseline
2	HIV treatment linkage at 1 year in women	% who visited the Department of Health clinics in the study area and initiated ART in women	Year 1
3	Population-level HIV viral suppression (both sexes)	Change in proportion with detectable viremia in both men and women; HIV testing and viral load measurements are performed on the DBS samples collected during the annual individual survey	Year 1
4	Population-level HIV viral suppression (both sexes)	Change in proportion with detectable viremia in both men and women; HIV testing and viral load measurements are performed on the DBS samples collected during the annual individual survey	Year 3
5	Population-level HIV viral suppression in women	Change in proportion with detectable viremia in women; HIV testing and viral load measurements are performed on the DBS samples collected during the annual individual survey	Year 1
6	Population-level HIV viral suppression in women	Change in proportion with detectable viremia in women; HIV testing and viral load measurements are performed on the DBS samples collected during the annual individual survey	Year 3
7	Population-level HIV viral suppression in men	Change in proportion with detectable viremia in men; HIV testing and viral load measurements are performed on the DBS samples collected during the annual individual survey	Year 3
8	Population level HIV incidence	Change in HIV incidence rate (number of HIV sero-conversions per 100 person-years of follow up) in both men and women; HIV testing and viral load measurements are performed on the DBS samples collected during the annual individual survey	Year 3
9	Population-level HIV-related mortality (both sexes)	Change in HIV-related mortality rate measured as the number of HIV-related deaths per 1000 person-years of observation in both men and women	Year 3
10	Population-level all-cause mortality (both sexes)	Change in all-cause mortality rate measured as the number of all-cause deaths per 1000 person-years of observation in both men and women	Year 3
11	Population-level HIV-related mortality in women	Change in HIV-related mortality rate measured as the number of HIV-related deaths per 1000 person-years of observation in women	Year 3
12	Population-level HIV incidence in men	Change in HIV incidence rate (number of HIV sero-conversions per 100 person-years of follow up) in men; HIV testing and viral load measurements are performed on the DBS samples collected during the annual survey	Year 3
13	HIV status knowledge in men and women	% ever received a test result for HIV in men and women	Year 1
14	HIV prevention knowledge in men and women	% reporting ever hearing about pre-exposure prophylaxis (PrEP)	Year 1
15	HIV treatment utilization in men and women	% reporting ever taking up ART for own health in men and women	Year 1
16	Reported condom use in men and women	% reporting condom use at last sex in men and women	Year 1
17	Tuberculosis (TB) healthcare utilization in men and women	% reported starting TB treatment in the past 12 months in men and women	Year 1
18	Diabetes Healthcare utilization in men and women	% reported starting diabetes treatment in the past 12 months	Year 1
19	Hypertension healthcare utilization in men and women	% reported starting blood pressure treatment in the past 12 months in men and women	Year 1
20	Household wealth (household assets)	Number of household assets (selected from a predefined list of 32 assets)	Year 3
21	Household wealth (food security)	% of adults in the household ever cutting the size of meals or missing meals due to insufficient money for food in the past 12 months	Year 3
22	Partnership patterns in men and women	% reporting having more than one sexual partner in the past 12 months in men and women	Year 1
23	Retention in HIV care in men and women	% who are retained in care (% patients having an ART clinic visit in previous 3 months) at 1-year post-initiation in men and women	Year 1
24	Retention in HIV care in men and women	% who are retained in care (% patients having an ART clinic visit in previous 3 months) at 3 years post-initiation in men and women	Year 3
25	Patient viral suppression in men and women	% who are remain virally suppressed (% patients where virus is undetectable) 1-year post-initiation in men and women	Year 1
26	Patient viral suppression in men and women	% who are remain virally suppressed (% patients where virus is undetectable) 3-years post-initiation in men and women	Year 3

### Data collection and measures

Participants are enrolled into the HITS trial during the ongoing annual individual HIV surveillance and routinely followed up between 2018 and 2021 (BREC Ref: BE290/16).

Trained fieldworkers visit participants at home once a year to conduct household and individual surveys using tablet-based computers. Data on demographic information, general health, and sexual behaviour (including contraceptive use, sexual activity and pregnancy history) are collected via the REDCap database [27]. Participating individuals are offered rapid HIV testing. Consenting individuals undergo confidential HIV pre- and post-test counselling according to the Department of Health procedures. A rapid HIV test is performed and test results are provided approximately 20 min after the testing. Participants who consent to the HIV surveillance have blood collected on a filter paper as DBS, which are stored at the AHRI Biorepository in Durban [28]. A 4th generation HIV ELISA (Genscreen™ ULTRA HIV Ag-Ab (Biorad, Marnes-la-Coquette, France) adopted and evaluated in-house by the AHRI Diagnostic Research Laboratory (unpublished data) is used for testing of all DBS samples. All HIV ELISA positive DBS samples are subjected to HIV viral load determination using the automated Nuclisens EasyMag® (Biomerieux, Marcy-l’Etiole, France). Viral load testing is performed on the same day as the extraction.

Individuals who test positive for HIV are referred to the nearby Department of Health clinics to receive HIV care within 10 days of the HIV test date. Data on linkage to care is collected from the Department of Health through TIER.net (i.e. an electronic patient records management system that contains information on all clinic visits for people on ART, including data for all patients attending the 17 clinics in the Hlabisa health sub-district and the Hlabisa hospital) and clinic attendance data (where date and reason for attendance are collected for all consenting individuals in the programme area who attend one of the 11 clinics serving the programme area). All deaths are ascertained by verbal autopsies with closest care givers using a questionnaire based on the INDEPTH/World Health Organization (WHO) standard questionnaire [29].

Data is collected and managed by AHRI Research Data management within the routine AHRI HIV and demographic surveillance study databases as per AHRI comprehensive study procedures. The database has strictly restricted access via a data enclave on a secure server.

### Randomisation and blinding

Randomisation was performed by the trial statistician prior to intervention rollout to ensure that each arm has similar baseline HIV incidence rates among women aged 15 and 30 years. First, all 45 clusters were stratified by HIV incidence in young women using data from 7605 young women aged 15 and 30 years old, who were residents in the PIPSA between 2006 and 2015 at least for 1 year. A total of 34551 person years and 1636 seroconversion events have been recorded, yielding overall 4.7 new infections per 100 person years. Then the 45 clusters were divided into four strata according to their HIV incidence rate such that stratum 1 comprises clusters with the lowest incidence rate, while stratum 4 comprises clusters with the highest incidence rate. Each of Arm 1, 2, and 3 consists of two clusters from each of the four incidence stratum (thus a total of eight clusters per arm). Arm 4 (SoC) consists of five clusters from each of the incidence stratum 1, 2, and 4, and six clusters from incidence stratum 3 (thus a total of 25-clusters). The study is an open label trial.

### Statistical methods

#### Power calculation

The study was powered to achieve a 25% reduction in HIV incidence among females aged 15–30 years old in the intervention arms. Using the AHRI’s actual HIV incidence data, we assumed HITS interventions were delivered in 2011 and simulated the effect of HITS interventions in 24 intervention communities after 3-years of follow up between 2012 and 2014. Using a simulation approach we then explored a scenario where the interventions lead to a 25% reduction in Arm 1 (micro-incentives), 25% reduction in Arm 3 (male-sensitive HIV specific counselling) and a 37% reduction in the combined arm (Arm 2) after 3-years of follow up.

**Fig. 5:**
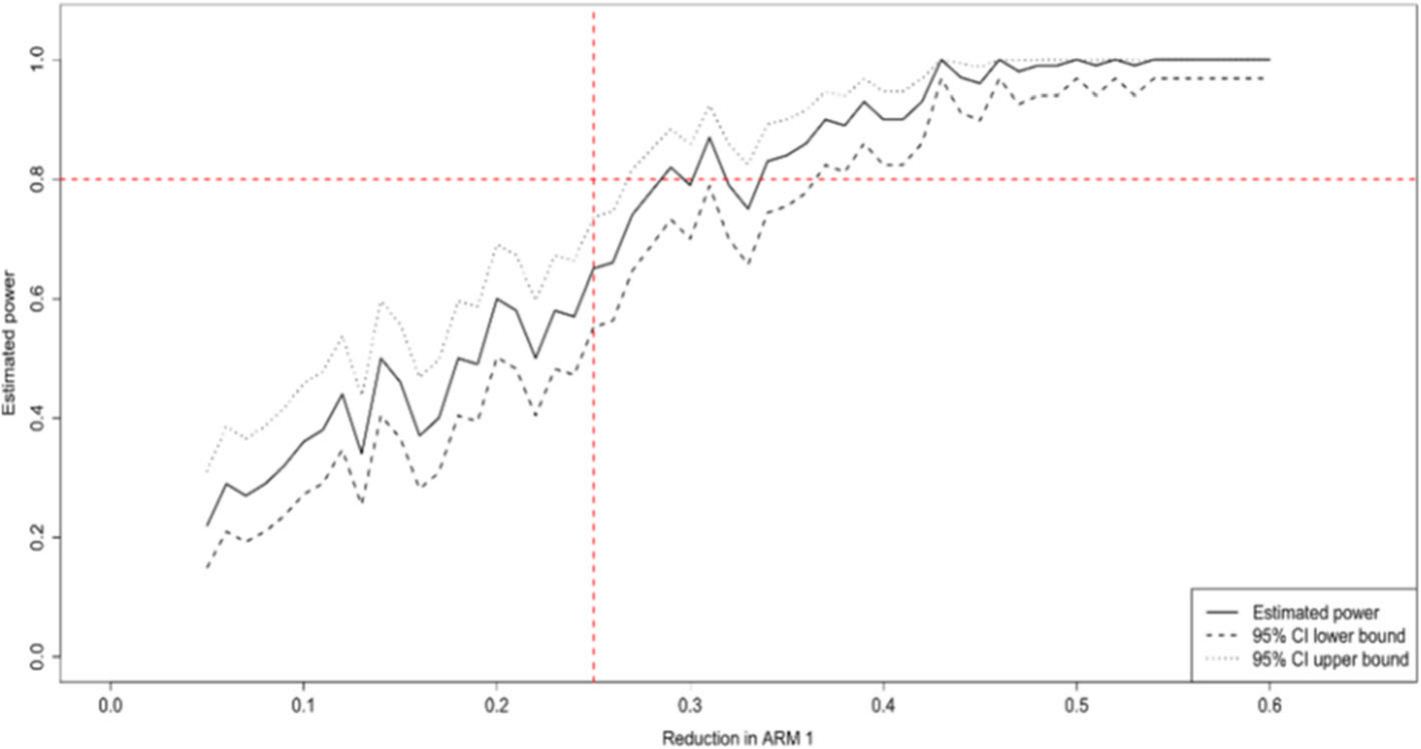
Power calculations. Example of data simulations to determine sample size required to detect a 25% decrease in HIV incidence in females aged 15–30 years (in Arm 1 or 3 relative to the SoC arm) using the AHRI population-based HIV surveillance data (2004–2014)

The results (Fig. 5) show that we would have been able to detect this reduction in incidence in > 80% of simulation replicates (*p* < 0.05). Therefore, if we were to introduce the HITS intervention in 2018 and follow young females up for at least 3 years post-intervention (i.e. utilize a total of 17 years of incidence data – 2004 to 2021), we would be in excess of 90% power to detect such a reduction in incidence in this critical age-group.

In the total of 24 communities in the intervention arms, an estimated total of 4,667 individuals will receive a HITS intervention. In the 21 communities, an estimated 4,900 individuals will receive the standard-of-care.

#### Statistical analysis

All primary analyses (both primary and secondary endpoints) will be intent-to-treat (ITT). For the binary primary outcomes – HIV testing uptake and HIV treatment linkage – we will use generalized linear models with Poisson distribution, log link function, and robust error terms to determine effect sizes (risk ratios). We will fit beta regression models for incidence and mortality rates [30, 31]. We will also adjust for both baseline endpoints and clustering through random effects. Under the difference-in-differences study design, the individuals are followed in two periods of time: the “before” and the “after” period, where the interventions occur in the “after” period [32]. The goal of the statistical analysis is to estimate the average treatment effect for the treated clusters (week blocks). We will make the usual assumption of parallel trend, that is, on average, the outcomes for all the clusters in the four arms would have followed a parallel path over time in the absence of the intervention [33]. Our generalized linear models will include fixed effects for the “before” and the “after” time periods, the grouping of the clusters in four arms, and the interaction between time periods and arms.

For the two survival-analytic primary endpoints – population-level mortality among men and population-level HIV incidence among women – we will use the Cox proportional hazards model to determine effect sizes (hazard ratios). If the proportional hazards assumption of the Cox model is violated, we will use appropriate alternative survival analytic models. We will use data from the period 2004–2017 for baseline endpoint adjustment. In addition, to the ITT analyses, we will measure intervention effects adjusted for non-compliance using instrumental variable (IV) approaches.

### Process evaluation

In order to determine how the implementation of the HITS intervention is achieved in our setting as well as the processes through which the interventions affect outcomes, we will gather stakeholder views in a purposive sample of up to 150 stakeholders (HITS participants, fieldworkers and health professionals) using mixed methods of quantitative and qualitative techniques (e.g. surveys and in-depth interviews). The evaluation is shaped by the UK Medical Research Council (MRC) guidance on the process evaluation of complex interventions [34]. The guidelines identify three important pathways of understanding the process of achieving a particular outcome: a) Context – we will examine how the socio-cultural context of our setting influences the development process, delivery and functioning of the HITS interventions; b) Implementation of the HITS program - we will describe the resources and processes through which HITS implementation is achieved, and the extent to which HITS intervention would be delivered as intended or needed any program adaptations; c) Mechanisms of impact - we will examine how study participants respond to and interact with HITS interventions and whether these interventions promote behavioural change or not. In order to examine the ‘causal assumptions’ of the intervention, the evaluation is informed by the Normalisation Process Theory (NPT) [35]. The theory will help us to understand how the HITS intervention would be delivered, as well as any changes that could affect its fidelity. All interviews will be recorded and transcribed and subject to thematic analysis following an interpretivist approach.

### Cost-effectiveness analysis

We will conduct a cost-effectiveness analysis of the two interventions, the micro-incentives and EPIC-HIV, from the societal perspective. A time and motion study will be conducted to determine how much time fieldworkers spend on the HITS related activities including consenting for study participation, HIV testing, counselling, and provision of micro-incentives and/or EPIC-HIV. Detailed information on direct and indirect costs will be documented during the study implementation to determine the unit costs of providing the interventions. Effectiveness will be measured as the number of uptake of HIV testing, new HIV diagnosis, as well as linkage to care. We will also fit mathematical modelling to simulate dynamic HIV transmission calibrated to the data from the trial and the AHRI surveillance and estimate disability-adjusted life years (DALYs) over 10 years. We will calculate the incremental cost-effectiveness ratio (ICER) for adding the interventions to standard of care.

## Discussion

The HITS trial will provide evidence for the impact and feasibility of micro-incentives and a male-sensitive HIV specific decision-support application to increase uptake of home-based HIV testing and linkage to HIV care, so as to reduce HIV-related mortality in men and population-level HIV incidence in young women in the hyperendemic rural South African setting. The study will also establish the mechanisms and the determinants of successful implementation through comprehensive process evaluation. Combination of quantitative and qualitative social science research produced from this study will be essential to understand the behavioural and social impact of offering micro-incentives and male-sensitive HIV specific decision support on the HIV care continuum and population-level health outcomes in this rural hyperendemic South African and other similar settings.

### Trial status

The study is ongoing.  Intervention delivery started in February 2018 and ended in December 2018. Participant follow-up will continue until the end of 2021. No articles containing the results of this study have been published or submitted for publication.

## Data Availability

The datasets for this study can be found upon request from Africa Health Research Institute (https://www.ahri.org). Investigators are required to outline use of the desired data with restricted access and sign a confidentiality and data access agreement. All other conditions need to be followed according to the AHRI’s protocol for data sharing.

## Abbreviations

AHRI: Africa Health Research Institute
ART: Antiretroviral Therapy
DBS: Dried Blood Spots
ELISA: Enzyme-Linked Immunosorbent Assay
EPIC-HIV: Empowering People through Informed Choices for HIV
GPS: Global Positioning System
HITS: Home-based Intervention to Test and Start
HIV: Human Immunodeficiency Virus
ITT: Intent to Test and Treat
MRC: Medical Research Council
NPT: Normalisation Process Theory
PIPSA: Population Intervention Program Southern Area
WHO: World Health Organization
